# Human iPSC-derived self-assembled cardiac organoids for evaluating drug developmental cardiotoxicity

**DOI:** 10.1093/lifemedi/lnaf018

**Published:** 2025-06-07

**Authors:** Yi Xiao, Pengfei Xu, Jinmiao Bi, Moshi Song

**Affiliations:** State Key Laboratory of Organ Regeneration and Reconstruction, Institute of Zoology, Chinese Academy of Sciences, Beijing 100101, China; Beijing Institute for Stem Cell and Regenerative Medicine, Beijing 100101, China; State Key Laboratory of Organ Regeneration and Reconstruction, Institute of Zoology, Chinese Academy of Sciences, Beijing 100101, China; Beijing Institute for Stem Cell and Regenerative Medicine, Beijing 100101, China; University of Chinese Academy of Sciences, Beijing 100049, China; State Key Laboratory of Organ Regeneration and Reconstruction, Institute of Zoology, Chinese Academy of Sciences, Beijing 100101, China; Beijing Institute for Stem Cell and Regenerative Medicine, Beijing 100101, China; University of Chinese Academy of Sciences, Beijing 100049, China; State Key Laboratory of Organ Regeneration and Reconstruction, Institute of Zoology, Chinese Academy of Sciences, Beijing 100101, China; Beijing Institute for Stem Cell and Regenerative Medicine, Beijing 100101, China; University of Chinese Academy of Sciences, Beijing 100049, China

## Dear Editor,

Drug cardiotoxicity is a significant concern in the development of congenital heart diseases, which are a leading cause of birth defects. Given the importance of drug safety in prenatal health, reliable models are essential for assessing potential cardiac developmental risks [1]. However, the differences in species-specific morphological and molecular characteristics limit the application of animal models in accurately assessing the cardiac teratogenicity and toxicity of drugs. Recognizing the gap, the US Food and Drug Administration (FDA) established a toxicity rating system in 1979. This system categorizes drug toxicity into five categories: Category A includes drugs proven safe in pregnant women with no increased risk of fetal abnormalities. Category B covers drugs not extensively studied in pregnant women but showing no harm in animal studies, or exhibiting adverse effects only in animals. Category C involves drugs not well studied in humans but showing adverse effects in animals, where benefits might outweigh risks. Category D includes drugs with demonstrated fetal risks in humans, where benefits might still outweigh risks. Lastly, Category X encompasses drugs with clear evidence of fetal abnormalities, where risks outweigh benefits [2]. Despite this classification, many drugs classified as low toxicity based on animal studies have been linked to heart developmental defects, causing structural and functional abnormalities in newborns. Thus, employing effective human cardiac models and conducting a more comprehensive evaluation of drug cardiac developmental toxicity are essential for preventing drug-related congenital heart diseases.

**Figure 1.**
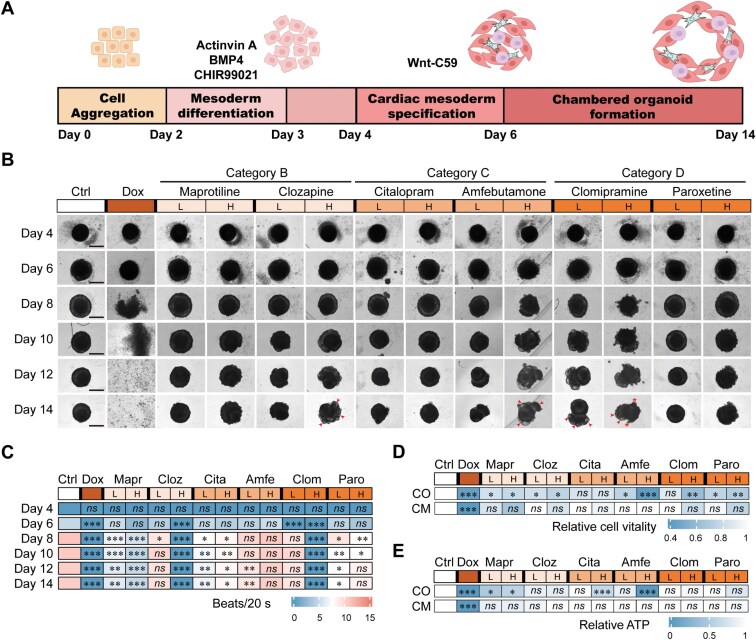
Generation and drug treatment of self-assembled cardiac organoids (COs). (A) Schematic diagram of the protocols to generate self-assembled COs from hiPSCs. Relevant growth factors and duration of differentiation steps are indicated. (B) Brightfield images of the development of organoids treated with lower (L, clinical-relevant) and higher (H, 10-fold higher) concentrations of drugs from Day 4 of organoid formation. The tested drugs were Maprotiline (L: 1 μM and H: 10 μM), Clozapine (L: 1 μM and H: 10 μM), Citalopram (L: 0.1 μM and H: 1 μM), Amfebutamone (L: 0.5 μM and H: 5 μM), Clomipramine (L: 1 μM and H: 10 μM), and Paroxetine (L: 0.15 μM and H: 1.5 μM). Vehicle of 0.1% DMSO (Ctrl) and 1 μM Dox were used as negative and positive controls, respectively. Scale bar, 200 μm. Arrows denote abnormal multicavity formation and stars denote abnormal spikes in COs at Day 14 of organoid formation. (C) Beating frequency of COs recorded over 20 s. *n* = 3 biological replicates each group. (D) Cell viability measured by Cell Counting Kit-8 (CCK8) in COs on Day 14 of organoid formation and hiPSC-derived cardiomyocytes (CMs) after treatment with indicated drugs for 10 days. Data were normalized to vehicle-treated control group. *n* = 3 biological replicates each group. (E) Measurement of ATP levels in CO on Day 14 of organoid formation and CM after treatment with indicated drugs for 10 days. Data were normalized to vehicle-treated CO and CM control groups. *n* = 3 biological replicates each group. Data are presented as mean ± SEM. Groups were compared using one-way ANOVA followed by post hoc Tukey’s test (C–E). *ns*, not significant; *P < 0.05; **P < 0.01; ***P < 0.001. Mapr, Maprotiline; Cloz, Clozapine; Cita, Citalopram; Amfe, Amfebutamone; Clom, Clomipramine; Paro, Paroxetine.

To develop a humanized assessment tool for cardiac developmental toxicity, we constructed self-assembled cardiac organoids from human induced pluripotent stem cells (hiPSCs) by regulating the activation and inhibition of the Wnt pathway, thereby recapitulating the early development of the heart. We evaluated the effects of a range of psychotropic drugs with distinct toxicity ratings (Fig. 1A). The tested drugs were introduced during cardiac mesoderm specification at both clinically relevant concentrations and 10-fold higher concentrations. Specifically, Category B drugs included Maprotiline and Clozapine; Category C drugs included Citalopram and Amfebutamone; and Category D drugs included Clomipramine and Paroxetine. Additionally, 0.1% DMSO was used as a negative control to account for solvent effects, while 1 μM Doxorubicin (Dox) served as a positive control due to its well-documented cardiotoxicity [2].

The morphology of the cardiac organoids was recorded daily from the stage of cardiac mesoderm specification to the formation of chambered organoids, spanning from Day 4 to Day 14 (Fig. 1B). Cardiac organoids treated with Dox exhibited dissociation and cell death as early as Day 8, indicating that these organoids are suitable for evaluating early cardiac developmental toxicity. Cardiac organoids treated with Maprotiline, Citalopram, and Paroxetine at both lower and higher doses, as well as lower doses of Clozapine and Amfebutamone, appeared largely normal. By contrast, abnormal multicavity formation was observed in organoids treated with higher doses of Clozapine and Amfebutamone. The fetal cardiotoxicity of Clomipramine, classified as Category D, was confirmed in the organoids. Specifically, abnormal multicavity formation was observed at both lower and higher doses, and the presence of spikes was noted at a higher dose (Fig. 1B), the latter of which likely indicates abnormalities in the cytoskeleton or contractile function of the cells.

Next, we systematically assessed the impact of these drugs on the beating rates of cardiac organoids from Day 4 to Day 14, uncovering distinct patterns of cardiac function modulation by different drugs (Fig. 1C). Most drugs induced a moderate to severe decrease in beating rates at higher doses, with the exception of Amfebutamone. At lower doses, Clozapine and Clomipramine had no significant effect on heart beating rates, whereas Amfebutamone and Paroxetine significantly reduced beating rates. Yet, the two drugs that significantly reduced beating rates to the greatest extent were Citalopram (Category C) and Maprotiline (Category B) (Fig. 1C), raising a cautionary note regarding their usage during early pregnancy. To further investigate whether the cardiotoxicity of the Category B drug Maprotiline was specific to the developing heart, we treated mature cardiac organoids with Maprotiline. There was no significant difference in beating frequency between the control and Maprotiline-treated mature cardiac organoids, suggesting that the observed cardiotoxicity of Maprotiline is indeed specific to the developing heart (Fig. S1).

Additionally, we evaluated cell viability and revealed that the majority of drug-treated groups exhibited significantly increased cell death compared to the vehicle-treated control groups on Day 14, except for Citalopram at both doses and Clomipramine at a lower dose (Fig. 1D). These data suggest that, despite largely normal structure, most of these drugs tested were able to cause damage at the cellular level. Additionally, we measured ATP levels in these organoids on Day 14. We found significant decreases in ATP levels in groups treated with higher doses of Citalopram and Amfebutamone, likely reflecting impaired cellular metabolic function by these drugs. Notably, significant decreases in ATP levels were observed in the Maprotiline-treated group at both lower and higher doses (Fig. 1E). In contrast, no significant differences were observed in hiPSC-derived cardiomyocytes treated with any of these drugs, regardless of dose, compared to vehicle controls (Fig. 1D and 1E). This suggests that the observed effects in our cardiac organoids may be specific to the early developmental stage of the human heart and that self-assembled cardiac organoids offer a more feasible platform for evaluating drug developmental cardiotoxicity.

**Figure 2.**
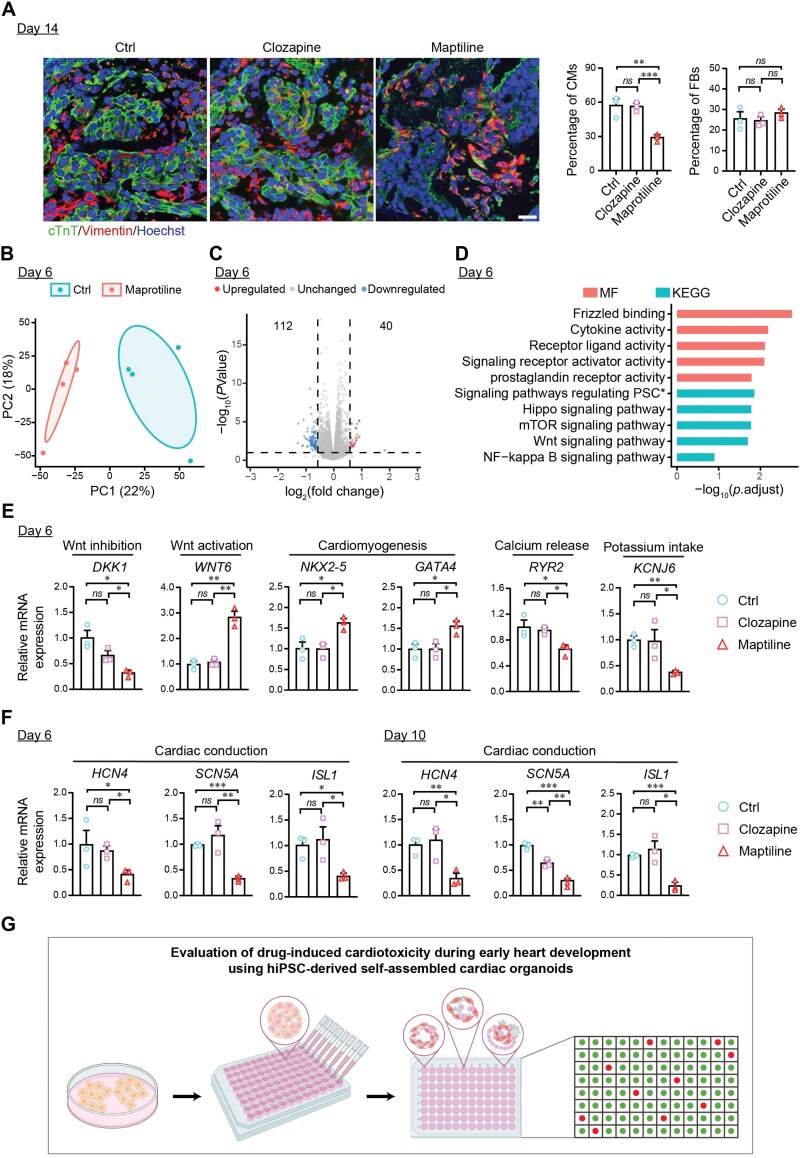
Maprotiline treatment interrupted developmental cardiomyocyte (CM) differentiation. (A) Representative immunofluorescence images and quantification of cardiac troponin T (cTnT)-positive CMs and Vimentin-positive fibroblasts (FBs) in cardiac organoids treated with Vehicle, 1 μM Maprotiline, and 1 μM Clozapine, starting from Day 4 of organoid formation and collected on Day 14. Cells were labeled with antibodies against cTnT (green) and Vimentin (red); nuclei were counterstained with Hoechst (blue). Scale bar, 20 μm. *n* = 3 biological replicates each group. (B) PCA of RNA sequencing data revealed distinct in-group clusters with minimal overlap between cardiac organoids treated with or without 1 μM Maprotiline, starting from Day 4 of organoid formation and collected on Day 6. *n *= 4 biological replicates each group. (C) Volcano plot illustrating differentially expressed genes (DEGs) identified between cardiac organoids treated with or without 1 μM Maprotiline. DEGs were identified with a cutoff of absolute log_2_(fold change) greater than 0.5 and a *P*-value less than 0.05. (D) GO Molecular Function (MF) and KEGG pathway enrichment analyses of the upregulated genes between cardiac organoids treated with or without 1 μM Maprotiline. PSC is short for pluripotency of stem cells due to the limited space in the figure. (E) qRT-PCR analysis of genes related to developmental CM differentiation in cardiac organoids treated with Vehicle, 1 μM Maprotiline, and 1 μM Clozapine, starting from Day 4 of organoid formation and collected on Day 6. Dickkopf Wnt signaling pathway inhibitor 1 (*DKK1*), Wnt Family Member 6 (*WNT6*), GATA binding protein 4 (*GATA4*), NK2 homeobox 5 (*NKX2-5*), ryanodine receptor 2 (*RYR2*), and potassium voltage-gated channel subfamily J member 6 (*KCNJ6*). *n* = 3 biological replicates each group. (F) qRT-PCR analysis of genes related to cardiac contraction in cardiac organoids treated with vehicle, 1 μM Maprotiline, and 1 μM Clozapine, starting from Day 4 of organoid formation and collected on Day 6 and Day 10. Hyperpolarization-activated cyclic nucleotide-gated potassium channel 4 (*HCN4*), sodium voltage-gated channel alpha subunit 5 (*SCN5A*), and ISL transcription factor 1 (*ISL1*). *n* = 3 biological replicates each group. (G) Schematic representation of a workflow for evaluating drug-induced cardiotoxicity during early heart development using hiPSC-derived self-assembled cardiac organoids. Data are presented as mean ± SEM. Groups were compared using one-way ANOVA followed by post hoc Tukey’s test (A, E, and F). *ns*, not significant; *P < 0.05; **P < 0.01; ***P < 0.001.

Notably, cardiac organoids treated with Maprotiline, a Category B drug, exhibited significant abnormalities in beating rates and ATP levels even at a lower dose. In contrast, cardiac organoids treated with Clozapine (also a Category B drug) at a lower dose maintained normal beating rates and ATP levels. To understand the impact of Maprotiline on cardiac organoids, we examined the changes in the cellular composition of the organoids using immunofluorescence staining. The results revealed that the Maprotiline-treated group had significantly fewer cardiomyocytes compared to the vehicle-treated control and Clozapine-treated groups (Fig. 2A). In contrast, the number of fibroblasts was comparable among these groups. This lower cardiomyocyte content likely explains the observed reduced beating rates and reduced ATP production, suggesting that Maprotiline may negatively influence developmental cardiomyocyte fate determination.

To elucidate the molecular mechanisms underlying Maprotiline’s effects on cardiac organoids, we conducted transcriptome analysis on both control and Maprotiline-treated organoids on Day 6 (Fig. 2B–D). Principal component analysis (PCA) revealed distinct differences between Maprotiline-treated and control organoids. In total, Maprotiline treatment resulted in the upregulation of 40 genes and the downregulation of 112 genes compared to the control (Fig. 2B and 2C). Gene Ontology (GO) and Kyoto Encyclopedia of Genes and Genomes (KEGG) enrichment analyses indicated that the upregulated genes were primarily associated with pathways involving frizzled binding (a Wnt receptor) and the Wnt signaling pathway (Fig. 2D). This is consistent with the well-established role of the Wnt signaling pathway in heart development, where precise temporal regulation is essential for proper cardiomyocyte specification.

Further, we performed qRT-PCR on genes involved in pathways related to Wnt inhibition and activation, as well as cardiomyocyte differentiation and maturation, on Day 6. We found that a Wnt inhibitor, Dickkopf Wnt signaling pathway inhibitor 1 (*DKK1*) [3], was significantly downregulated, and a Wnt ligand, Wnt Family Member 6 (*WNT6*) [3], was significantly upregulated by Maprotiline treatment compared to the vehicle-treated control and Clozapine-treated group. This indicates continuous Wnt activation by Maprotiline treatment, which should have been downregulated at Day 6 (Fig. 2E). Consistently, the cardiac progenitor cell marker genes, GATA binding protein 4 (*GATA4*) and NK2 homeobox 5 (*NKX2-5*) [4], were significantly upregulated, while a regulator of cardiac excitation-contraction coupling and intracellular calcium homeostasis, ryanodine receptor 2 (*RYR2*) [5], and a potassium voltage-gated channel subfamily J member 6 (*KCNJ6*) [6], were both significantly downregulated only in the Maprotiline-treated group (Fig. 2E). These data suggest that Maprotiline treatment causes continuous activation of the Wnt pathway and thus blocks cardiomyocyte differentiation and maturation.

Lastly, we evaluated the expression of genes related to cardiac contraction at both Day 6 and Day 10 of organoid formation. The results showed that cardiac conduction-related genes, including hyperpolarization-activated cyclic nucleotide-gated potassium channel 4 (*HCN4*) [7], sodium voltage-gated channel alpha subunit 5 (*SCN5A*) [8], and ISL transcription factor 1 (*ISL1*) [9], were decreased at both Day 6 and Day 10 (Fig. 2F), further supporting the negative impact of Maprotiline on developmental cardiomyocyte differentiation and maturation. By contrast, in hiPSC-derived cardiomyocytes, the expression of *HCN4* and *SCN5A* was unaffected by Maprotiline compared to vehicle-treated and Clozapine-treated groups (Fig. S2A and S2B). These findings further underscore the negative impact of Maprotiline on developmental cardiomyocyte fate determination and highlight the efficacy of using hiPSC-derived self-assembled cardiac organoids for evaluating drug developmental cardiotoxicity.

Collectively, our study utilizes hiPSC-derived self-assembled cardiac organoids to evaluate drug developmental cardiotoxicity and reveals the negative impact of Maprotiline on developmental cardiomyocyte fate determination. Importantly, this study highlights the potential of hiPSC-derived self-assembled cardiac organoids as a reliable and human-relevant model for assessing drug developmental cardiotoxicity, offering a more accurate alternative to traditional animal models, potentially preventing drug-induced congenital heart diseases and improving prenatal health.

## Research limitation

Although this study assessed the cardiotoxicity of multiple psychotropic drugs on early cardiac development using a hiPSC-derived self-assembled cardiac organoid model, broader validation across diverse pharmacological classes is needed to establish the generalizability of the model. Additionally, the current self-assembled organoid construction method relies predominantly on manual techniques, which impedes scalability and limits its applicability for high-throughput drug toxicity screening [10] . To overcome these constraints, future research should prioritize the development of standardized, automated, and scalable manufacturing processes to enhance reproducibility and accelerate the integration of organoid models into large-scale preclinical workflows.

## Supplementary Material

lnaf018_suppl_Supplementary_Figures_S1-S2
