# Supplementary material for: Human iPSC-derived self-assembled cardiac organoids for evaluating drug developmental cardiotoxicity
Source: Life Med. 2025 Jun 7;4(4):lnaf018. doi: 10.1093/lifemedi/lnaf018 (PMC12238535; doi:10.1093/lifemedi/lnaf018)
Supplement: lnaf018_suppl_Supplementary_Figures_S1-S2 [file lnaf018_suppl_supplementary_figures_s1-s2.docx]

**Human iPSC-derived self-assembled cardiac organoids for evaluating drug developmental cardiotoxicity**

Yi Xiao^1,2^, Pengfei Xu^1,2,3^, Jinmiao Bi^1,2,3^, Moshi Song^1,2,3,*^

^1^State Key Laboratory of Organ Regeneration and Reconstruction, Institute of Zoology, Chinese Academy of Sciences, Beijing 100101, China

^2^Beijing Institute for Stem Cell and Regenerative Medicine, Beijing 100101, China

^3^University of Chinese Academy of Sciences, Beijing 100049, China

^*^Correspondence: songmoshi@ioz.ac.cn

**Supplemental figures and figure legends**


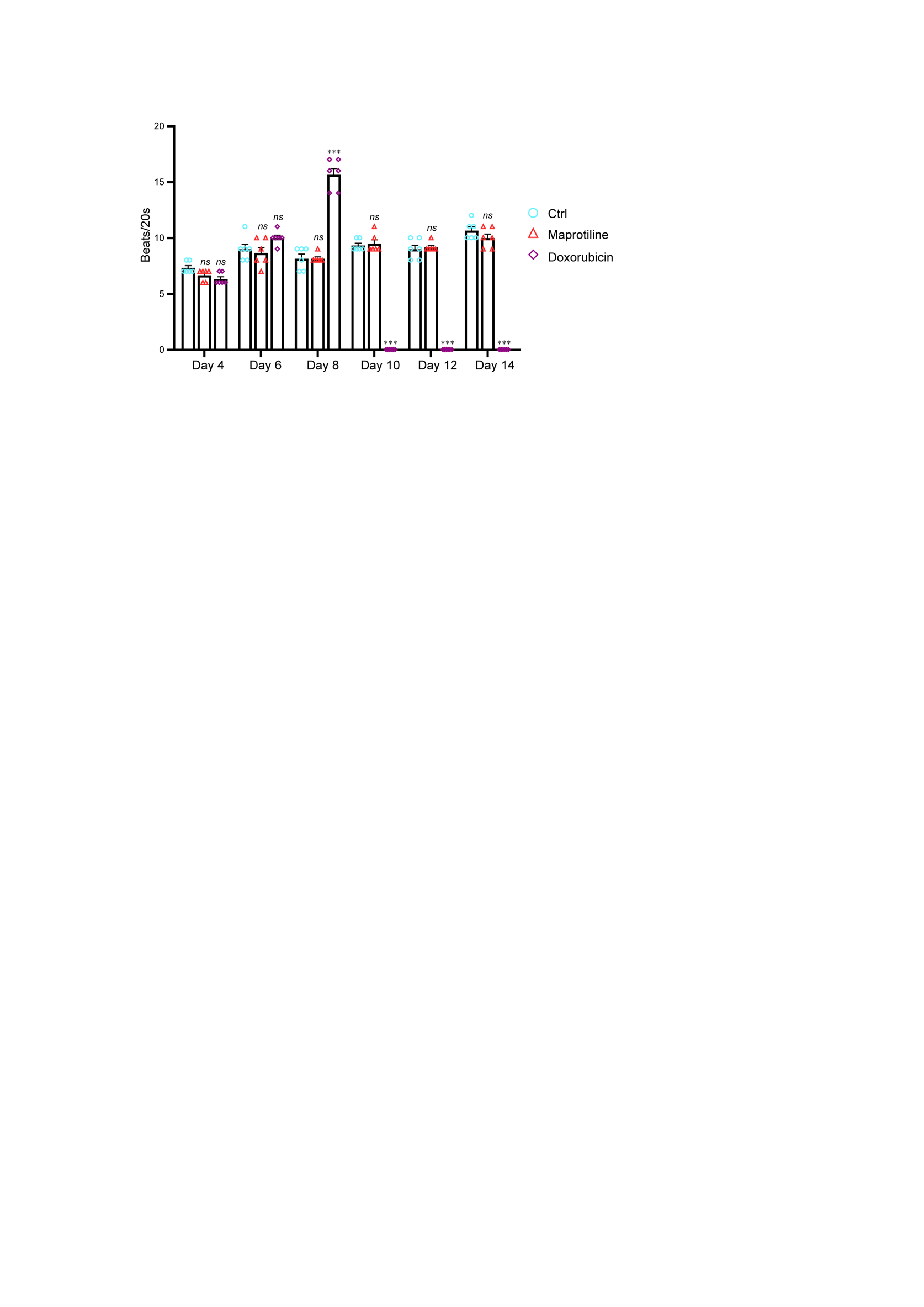


**Figure S1. Maprotiline exhibited no adverse effect on beating in mature cardiac organoids.**

Beating frequency of mature cardiac organoids treated with 0.1% DMSO, 1 μM Maprotiline, and 1 μM Doxorubicin from Day 4 to Day 14. *n* = 6 biological replicates each group. Data are presented as mean ± SEM. Groups were compared using Student’s *t*-test. *ns*, not significant; ****p* < 0.001.


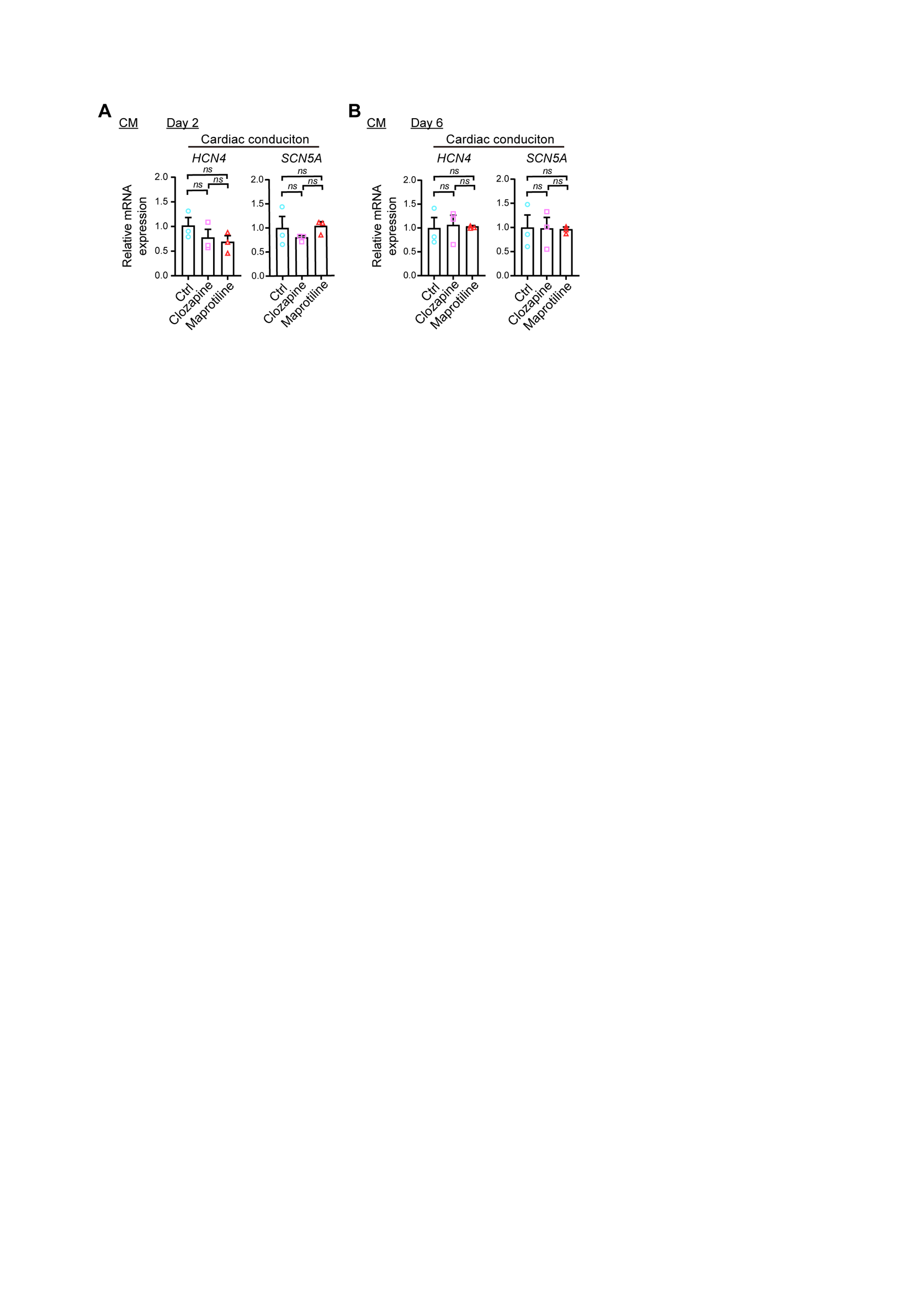


**Figure S2. Maprotiline exhibited no adverse effect on cardiac conduction gene expression in hiPSC-derived cardiomyocytes (CMs).**

(A, B) qRT-PCR analysis of genes related to cardiac contraction in hiPSC-derived CMs treated with vehicle, 1 μM Maprotiline, and 1 μM Clozapine for 2 days (A) and 6 days (B). Hyperpolarization-activated cyclic nucleotide-gated potassium channel 4 (*HCN4*) and sodium voltage-gated channel alpha subunit 5 (*SCN5A*). *n* = 3 biological replicates each group. Data are presented as mean ± SEM. Groups were compared using one-way ANOVA followed by post hoc Tukey’s test. *ns*, not significant.

**Methods**

**Maintenance of human induced pluripotent stem cells**

The human induced pluripotent stem cell (hiPSC) line provided by the National Stem Cell Resource Center (NSCRC), Institute of Zoology, Chinese Academy of Sciences was derived from amniotic mesenchymal cells (Ethics Committee Approval: Institute of Zoology, Chinese Academy of Sciences, China; Cell Line: Q-iPS-6, permit number: DYSL[2023]004). The hiPSCs were cultured in chemically defined PSCeasyII medium (Cellapy, CA1014500) in an incubator at 37°C with 5% CO₂ until they reached 80% confluency. The cells were dissociated using 0.5 mM EDTA and passaged at a split ratio ranging from 1:10 to 1:20. For replating, the cells were seeded onto plates coated with 0.5% Matrigel (Corning, 356230) in PSCeasyII medium supplemented with 10 μM ROCK inhibitor Y-27632 (Selleck, S1049) for the first day. From the second day onwards, the cells were maintained in PSCeasyII medium alone until they reached 80% confluency for downstream applications.

**Formation of self-assembled cardiac organoids**

Self-assembled cardiac organoids were generated following a previously published protocol^1^ with slightly modifications. On Day 0, hiPSCs were dissociated using 0.5 mM EDTA and resuspended in PSCeasyII medium containing 2 μM Y27632. The cell suspension was adjusted to a concentration of 10^5^ cells/mL and seeded at 100 μL per well in round-bottom ultra-low attachment 96-well plates (Corning, 7007). The plates were centrifuged at 100 *g* for 3 min and incubated for one day. On Day 1, the medium was replaced with 200 μL of PSCeasyII medium without Y27632. On day 2, the medium was replaced with 200 μL of RPMI-1640 (Gibco, 11875-093) supplemented with B27 minus insulin (Gibco, A1895601) (RPMI+B27-ins), containing 1 ng/mL Activin A (STEMCELL Technologies, 78001), 1.25 ng/mL BMP4 (STEMCELL Technologies, 78211), and 4 μM CHIR99021 (Selleck, S2924). On Day 3, the medium was replaced with 200 μL of RPMI+B27-ins medium alone. On Day 4, 2 μM Wnt-C59 (Selleck, S7037) was added to the medium for two days. From Day 6 onwards, the medium was changed to 200 μL of fresh RPMI+B27-ins medium every other day. Images of cardiac organoids were captured using Cytation 5 (Agilent, USA).

**Drug treatment**

All drugs were purchased from Selleck at a concentration of 10 mM in DMSO. The drugs used included Maprotiline (S2517), Clozapine (S2459), Citalopram (S4749), Amphetamine (S2452), Clomipramine (S2541), and Paroxetine (S3005). 0.1% DMSO was used as a negative control and 1 μM Doxorubicin served as a positive control for cardiotoxicity. For cardiac organoids, the tested drugs were added at the indicated concentrations starting from Day 4 of cardiac organoid formation and were replenished with medium changes every other day. For hiPSC-derived cardiomyocytes, the tested drugs were added for two days prior to downstream analyses.

**Generation of hiPSC-derived cardiomyocytes**

HiPSCs were cultured to 80% confluence in PSCeasyII medium and were treated with RPMI+B27-ins medium and 4 μM CHIR99021 for two days. The cells were then cultured in RPMI+B27-ins medium for an additional day upon the removal of CHIR99021. Subsequently, on Day 3, cells were treated with RPMI+B27-ins medium and 5 μM Wnt pathway inhibitor IWR-1 (Selleck, S7086) for two days. From Day 5 onwards, the cells were maintained in RPMI+B27-ins medium, with the medium changed every other day. On Day 11, cells were cultured in glucose-free RPMI (Gibco, 11879020) medium supplemented with B27 minus insulin (Gibco, A1895601) for 4 days, with the medium changed every other day, to induce glucose starvation for cardiomyocyte purification. Starting from Day 15, the purified cardiomyocytes were maintained in RPMI+B27-ins medium, with the medium changed every other day, until subsequent experiments were conducted around Day 30.

**Formation of mature cardiac organoids**

On day 20 of hiPSC differentiation, the cardiomyocytes were dissociated using 2.5% TryPLE (Gibco, 12605010) at 37°C. After centrifugation, the cardiomyocytes were resuspended in RPMI+B27-ins with 10 μM Y-27632 at a concentration of 2.5×10^5^ cells/mL and seeded into a 96-well ultra-low-adherence plate (Corning, 7007) with 200 μL per well as day 0. After one day, the medium was replaced with fresh RPMI+B27-ins. 0.1% DMSO, 1 μM Doxorubicin, and 1 μM Maprotiline were added at from Day 4 of cardiac organoid formation and were replenished with medium changes every other day.

**Measurement of beating frequency**

Beating frequency of cardiac organoids was recorded over a 20-second interval using Cytation 5 (Agilent, USA) every other day from day 4 to day 14. The results were presented as heatmaps visualized using the pheatmap R package (version 1.0.12).

**Cell viability assessment**

Cell Counting Kit-8 (CCK8) reagent (Beyotime, C0037) was used to assess cell viability of cardiac organoids and hiPSC-derived cardiomyocytes following the manufacturer’s instructions. The signals were detected by absorbance at 450 nm using a Cytation5 multi-well plate reader (Agilent, USA). The results were presented as heatmaps visualized using the pheatmap R package (version 1.0.12).

**Detection of ATP levels**

Cardiac organoids and hiPSC-derived cardiomyocytes were lysed and detected using an ATP Assay Kit (Beyotime, S0026) following the manufacturer’s instructions. The signals were detected using a Cytation5 multi-well plate reader (Agilent, USA) with normalization to protein content determined by the BCA Protein Assay Kit (Beyotime, P0012). The results were presented as heatmaps visualized using the pheatmap R package (version 1.0.12).

**Immunofluorescence staining of cardiac organoids**

Cardiac organoids were fixed in 4% paraformaldehyde (PFA) at 4°C overnight. They were then subjected to a gradient dehydration process using 15% and 30% sucrose solutions, followed by embedding in 20% gelatin to facilitate frozen sectioning at a thickness of 5 μm. Antigen retrieval was performed using a microwave oven with citric acid buffer. Subsequently, the sections were permeabilized and blocked with QuickBlock™ Blocking Buffer for Immunol Staining (Beyotime, P0260) for 1 hr at room temperature. The sections were then incubated with primary antibodies at 4°C overnight. The following day, the sections were rinsed with PBS and incubated with Alexa-Fluor-conjugated secondary antibodies and Hoechst 33342 (Beyotime, C1022) for 1 hr at room temperature. Immunofluorescence images were captured using an Andor Dragonfly 505 microscope, and the signals were analyzed using ImarisViewer (x64 9.6.0) software.

Primary antibodies used were mouse anti-cTnT (Abcam, Ab10214) and rabbit anti-Vimentin (Proteintech, 10366-1-AP). Secondary antibodies used were donkey anti-rabbit conjugated with Alexa Fluor™ 488 (Thermo Fisher Scientific, A-21202) and goat anti-rabbit conjugated with Alexa Fluor™ 568 (Thermo Fisher Scientific, A-11011).

**RNA sequencing and data analysis**

Total RNA was extracted from cardiac organoids using the FastPure Cell/Tissue Total RNA Isolation Kit V2 (Vazyme, RC112-01). Sequencing libraries were generated using NEBNext Ultra RNA Library Prep Kit for Illumina (NEB, E7530L) following the manufacturer’s instructions. Qualified libraries were sequenced on Illumina platforms using a paired-end 150 (PE150) strategy (Novogene Bioinformatics Technology). With variance stabilizing transformation of the read counts, PCA was performed using the prcomp function of the R Stats Package (version 3.6.2). Differentially expressed genes (DEGs) were identified using the DESeq2 software package (version 1.40.2) with a Negative Binomial Generalized Linear Model (NB-GLM) for statistical inference. DEGs were defined by setting simultaneous thresholds for an absolute value of log2(fold change) greater than 0.5 and a *p*-value less than 0.05, with multiple testing correction performed using the Benjamini-Hochberg method. Volcano plots were generated using the ggplot2 package (version 3.4.2). DEGs were then subjected to Gene Ontology (GO) Molecular Function (MF) and Kyoto Encyclopedia of Genes and Genomes (KEGG) pathway enrichment analyses using the clusterProfiler R package (version 4.8). The results of these analyses were visualized with ggplot2 (version 3.4.2) using a hypergeometric test to assess the overrepresentation of functional categories and pathways among DEGs. Multiple testing correction was applied using the Benjamini-Hochberg method, with an adjusted *p*-value threshold of 0.05 to determine significantly enriched terms.

**qRT-PCR analysis**

Total RNA was extracted from cardiac organoids or hiPSC-derived cardiomyocytes using the FastPure Cell/Tissue Total RNA Isolation Kit V2 (Vazyme, RC112-01) and reverse-transcribed into cDNA using the RevertAid™ First Strand cDNA Synthesis Kit (Thermo Scientific, M1632). qPCR analysis was performed using the Taq Pro Universal SYBR qPCR Master Mix (Vazyme, Q712-03). The *18S* gene was used as a reference gene for normalization, and data were analyzed using the 2^−ΔΔ^*^Ct^* method.

The sequences of the primers used for qPCR are listed below:

*DKK1* Fw: 5'-TGACAACTACCAGCCGTACC-3';

*DKK1* Re: 5'-CAGGCGAGACAGATTTGCAC-3';

*WNT6* Fw: 5'- CGATGCTGCCGCCCTTA-3';

*WNT6* Re: 5'- CTGCCCACAGCCCACC-3';

*GATA4* Fw: 5'- CCAGGCCCAGCAGATATGTTT-3';

*GATA4* Re: 5'- GCACAGATAGTGACCCGTCC-3';

*NKX2*-*5* Fw: 5'-GAGCCGAAAAGAAAGGGTGTG-3';

*NKX2-5* Re: 5'-CACCGACACGTCTCACTCAG-3';

*RYR2* Fw: 5'- CTCTTCTGGATGACCGAACCA -3';

*RYR2* Re: 5'- TTCGGCTCTGGCTGCATGAT -3';

*KCN6J* Fw: 5'- CTCGCCATCCGTCGTGTAGT-3';

*KCN6J* Re: 5'- CTTCTTTGTGCTTTTCCTGGCT-3';

*HCN4* Fw: 5'-GCGAACAGGAGAGGGTCAAG-3';

*HCN4* Re: 5'-GTCAGGTCCCAGTAAAATCTGA-3';

*SCN5A* Fw: 5'-GAGGCCAGTGCATCTCAGG-3';

*SCN5A* Re: 5'-AACTGTCCTCTGGGGTCTCA-3';

*ISL1* Fw: 5'-CATGTTTGAAATGTGCGGAGTG-3';

*ISL1* Re: 5'-GCATTTGATCCCGTACAACCTGATA-3';

*18S* Fw: 5'-GCTTAATTTGACTCAACACGGGA-3';

*18S* Re: 5'-AGCTATCAATCTGTCAATCCTGTC-3'.

**Statistical analysis**

All statistical analyses were performed using GraphPad Prism 10.3 software (Prism Inc., San Diego, CA, USA). Statistical comparisons between the two independent groups were conducted using a two-tailed unpaired Student’s *t*-test or one-way analysis of variance (ANOVA) followed by post hoc Tukey’s test as appropriate. Data are presented as mean ± SEM. Differences are considered statistically significant at *p* < 0.05.

Data availability

RNA sequencing data have been deposited in the Genome Sequence Archive in National Genomics Data Center, China National Center for Bioinformation (GSA-Human: HRA010185) that are accessible at https://ngdc.cncb.ac.cn/gsa-human.

**Reference**

1 Lewis-Israeli, Y. R. *et al.* Self-assembling human heart organoids for the modeling of cardiac development and congenital heart disease. *Nat Commun* **12**, 5142, doi:10.1038/s41467-021-25329-5 (2021).
